# Recent technological innovations in mycelium materials as leather substitutes: a patent review

**DOI:** 10.3389/fbioe.2023.1204861

**Published:** 2023-08-07

**Authors:** Elise Elsacker, Simon Vandelook, Eveline Peeters

**Affiliations:** Research Group of Microbiology, Department of Bioengineering Sciences, Vrije Universiteit Brussel, Brussels, Belgium

**Keywords:** leather-like mycelium materials, biomaterial patents, fungal fermentation, filamentous fungi, crosslinking

## Abstract

Leathery mycelium materials, made from the vegetative part of filamentous fungi, have garnered significant interest in recent years due to their great potential of providing environmentally sustainable alternatives to animal- and plastic-based leathers. In this systematic patent review, we provide an in-depth overview of the fabrication methods for mycelium materials as leather substitutes recently described in patents. This overview includes strategies for fungal biomass generation and industrial developments in the sector. We discuss the use of various fungal species, plasticizers, crosslinking agents, and post-processing techniques, thereby highlighting potential gaps in scientific knowledge and identifying opportunities, challenges, and concerns in the field. Our analysis suggests that mycelium materials have significant potential for commercialization, with a growing number of companies betting on this new class of biomaterials. However, we also reveal the need for further scientific research to fully understand the properties of these materials and to unlock potential applications. Overall, this patent review delineates the current state of the art in leathery mycelium materials.

## 1 Introduction

As society looks for environmentally conscious solutions to tackle issues related to ecological destruction and resource scarcity by seeking to broaden the sustainable material base, researchers are turning to biology for inspiration in the design and engineering of advanced materials. One area of growing interest is the development of mycelium materials, which are made from the vegetative part of filamentous fungi. These materials can be grown on a wide variety of agricultural and industrial organic waste- or side streams, which has led to a burgeoning interest in experimentation with new mycelium species and the design of new fermentation setups. Many industries, from construction to chemicals and textiles manufacturers, are pressured towards biobased and circular economy strategies due to consumer demand, evolving environmental regulations, and industry-imposed targets, resulting in a rising interest in biomaterials. In addition, mycelium materials can offer a relative low-cost and environmentally sustainable alternative to some petroleum-based materials (Stelzer et al., 2021; Livne et al., 2022; Williams et al., 2022).

While the initial focus was placed on the production of lignocellulosic mycelium composites, generating dense or semi-dense solid materials with potential application in the construction and packaging industries (Jones et al., 2017; 2020b; Abhijith et al., 2018; Elsacker et al., 2020), a recent shift in interest has occurred towards the development of new processes in which the fungus is grown as a biological tissue or mat on top of a liquid and/or solid substrate, or as fungal biomass in submerged liquid fermentation (Jones et al., 2020a; Gandia et al., 2021b; Vandelook et al., 2021). These materials consist mainly of fungal biomass, have textile-, leather- or foam-like properties and can display functionalities as a leather-like substitute material (e.g., clothes, bags, and seat covers). There is an increasing need for environmentally friendly alternatives because traditional leather production has its limitations. Leather is a by-product of the animal farming business, which ties it to an industry responsible for a large carbon footprint as well as other ecotoxicities (water pollution, human health, and land-use impacts) and the intensive use of hazardous chemicals in the hide tanning process. Conversely, plastic-based leathers, such as “PU leather” or “vegan leather”, have a lower carbon footprint than animal leather during their production, but they are dependent on fossil resources and have negative environmental effects (microplastic pollution, landfill, and ocean accumulation). Consequently, the increasing demand for sustainable materials has led to the development of “alternative leather” technologies based on mycelium and various other organic streams (e.g*.*, Piñatex, Vegea or Fruitleather).

The recent release of multiple prototypes of leathery mycelium materials is indicative for a maintained commercial interest. Since 2019, there is a clear visible increase in patent filings on mycelium materials, fungal fermentation technologies and functionalization strategies, aimed towards the commercialization of animal and synthetic leather substitutes. Consequently, a new ecosystem of companies betting on mycelium is emerging, following in the footsteps of early adopters and current industry leaders like Ecovative, Mycowork, and Mogu. This has led to aggregated knowledge clusters in the patent landscape, which tends to focus on the effectiveness and useability of the technology in frame with the economic value, rather than on the generation and dissemination of a significant body of scientific knowledge and data.

In this systematic review, the primary objective is to analyze the patent landscape in the field of fungal-based leather-like materials and to provide insights into the innovative technologies and approaches used in the fabrication of fungal materials as leather substitutes. We discuss recent trends in fungal fermentation techniques and industrial developments in the sector. An overview is provided of the use of various fungal species, plasticizers, crosslinking agents, and post-processing techniques. We also identify potential gaps in scientific knowledge, as well as opportunities, challenges, and concerns in the field. By focusing primarily on patent literature, our aim is to offer an application-focused review of the advancements made in the industrial sector of mycelium materials. Due to the scope of this paper, we do not include a broader set of references from the scientific literature. Interested readers are therefore directed to existing literature reviews for further information (Jones et al., 2020a; Gandia et al., 2021b; Vandelook et al., 2021; Peeters et al., 2023).

## 2 Methods

### 2.1 Patent search

The Espacenet website was used to search for patents based on a keyword search approach in the patent titles and abstracts. The following combinations of keywords were used: “mycelium” and “leather”, or “mycelium” and “mat”, or “fungal” and “leather”, or “fungal” and “mat”, or “mycelium” and “material”, or “non-woven” and “mycelium”, or “mycelium” and “textile”, or “mycelium” and “flexible” and “material”. The search was focused on the IPC class C12N1/14, which is used for fermentations with fungi. The time interval was limited to patents filed or granted in the period from 2009 to 2023. Results were screened for relevance by manually reading the abstracts or entire patents, omitting patents concerning solid mycelium composites, food or medical applications. The remaining patents were additionally validated with Google Patents to analyze the chronology of events and the countries where they were granted. The patent search was conducted in February 2023 and a total of 36 patents were selected that cover mycelium leather-like materials. Patents that were not included in the Espacenet database or patents that did not mention the above keywords in the title or abstract or that were abandoned by the applicants, were not used in this study.

## 3 Results

### 3.1 Overview of patents on mycelium leather-like materials

In this study, a selection of 36 patents was made that cover fungal-derived materials with the intended application as a leather substitute or as a textile or fabric. Patents relating to the use of rigid mycelium composites (Cerimi et al., 2019) or fruiting bodies to make amadou and other related materials are not addressed (Gandia et al., 2021b).

Interestingly, two patents were already granted to the applicants Ford and Ecovative in 2011 and 2012. Although the methods provided do not specifically focus on a leather replacement use, as all patents beginning in 2019 do, these two patents address the production of mycelium mats. Ecovative’s patent, in particular, details how mycelium would naturally grow over the surface of a nutrient-rich fluid, solid, or solid-liquid boundary (woven or matt fiber atop nutritional broth) and how it may be harvested for thin film applications (Mcintyre et al., 2012). Afterwards, these mycelium sheets can be processed (cut, pressed) to graft desired two-dimensional characteristics on individual sheets (Mcintyre et al., 2012).

**FIGURE 1:**
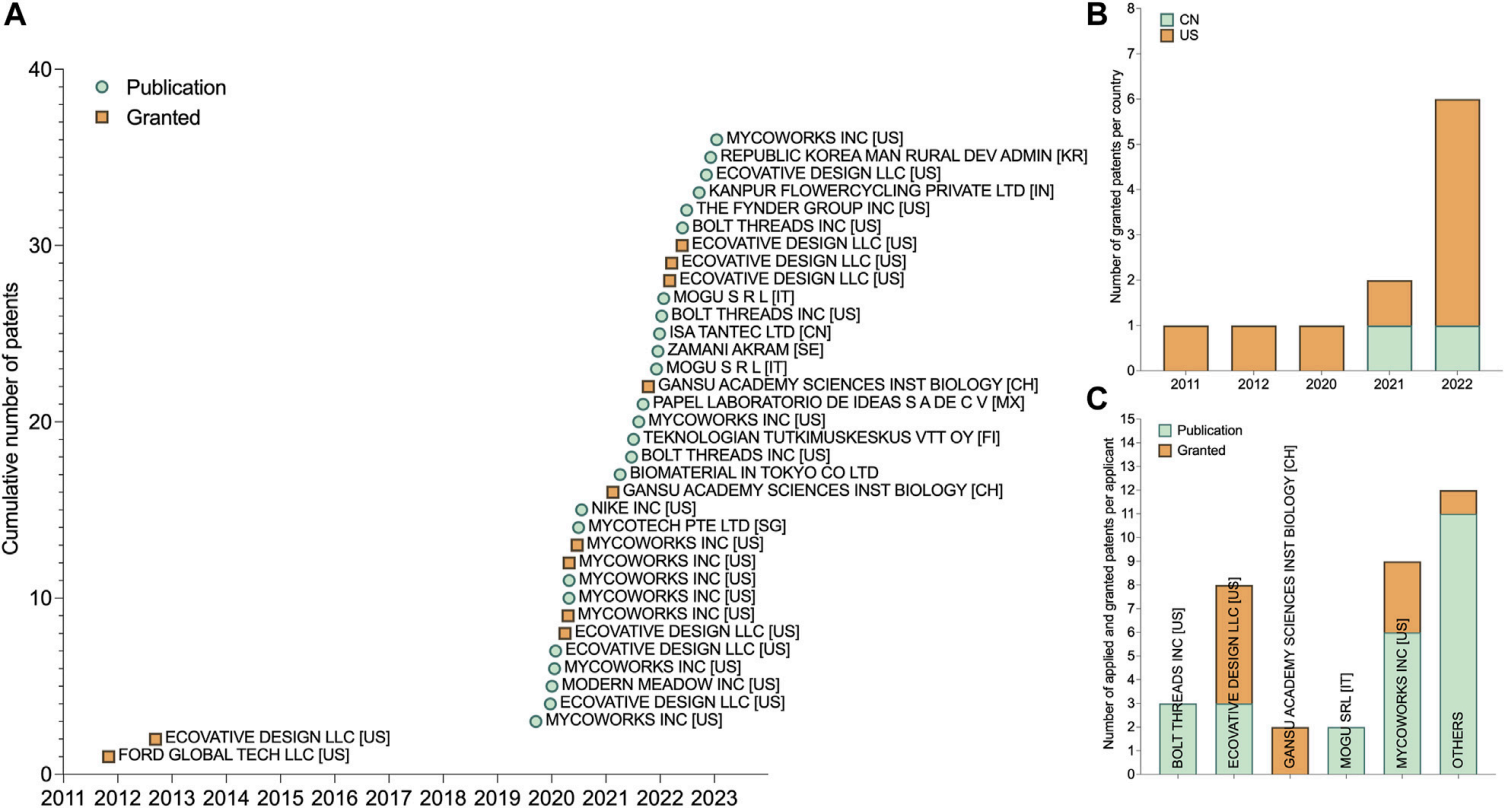
Patents on mycelium leather-like materials. **(A)** Cumulative number of patents published per year. **(B)** Distribution of granted patents over the years per country. Country codes: US (United States); CN (China). **(C)** Published and granted patents per applicant.

Starting at the end of 2019, the number of patent filings describing mycelium leather-like materials has increased considerably (Figure 1A). As of today, only 11 of the 36 relevant patents have been granted, with the majority going to applicants in the United States. With a total of 25 remaining pending applications, it is likely that the number of granted patents on mycelium leather-like materials will continue to increase in the near future (Figure 1B). Ecovative (5 granted and 3 pending applications) and Mycoworks (3 granted and 6 pending applications) now hold the bulk of patents, followed by the Chinese academic institute Gansu Academy Sciences Institute Biology (2 granted applications) (Figure 1C). The remaining patent applications, which are mostly pending, are divided among different companies, each of which has one or two applications.

### 3.2 Determinants in inventions with leather-like mycelium materials

#### 3.2.1 Fungal species

When describing a material as being a mycelium-based material, it is expected that the majority of the material’s composition is derived from fungal biomass. Therefore, the choice of the fungal species can significantly influence the production process and the final material properties by means of their biological characteristics. The examination of which species were mentioned in the selected patents involved a meticulous analysis of both the claims and the descriptions provided within each patent. Fungal species were either explicitly stated in the claims or were only listed as examples without additional information in the claims section.

According to the hereabove mentioned elements, a total of 69 organisms were identified across granted and non-granted patents (Table 1). *Ganoderma* (mentioned in 5 patents) and *Trametes* (mentioned in 4 patents) are the most commonly listed genera in patent descriptions, followed by *Fomes*, *Fusarium*, *Pleurotus*, and *Schizophyllum* (each mentioned in 3 patents). Except for *Fusarium*, which is an Ascomycete, all of the above-mentioned species are members of the Basidiomycetes.

**TABLE 1:** List of species mentioned patents to produce mycelium leather substitutes.

Genus	Species	References
*Agaricus*		Smith et al. (2021), Stewart et al. (2021)
	*Agaricus arvensis*	Smith et al. (2021)
	*Agaricus bisporus*	Stewart et al. (2021)
*Agrocybe*		Smith et al. (2021)
	*Agrocybe brasiHensis*	Smith et al. (2021)
*Amylomyces*		Smith et al. (2021)
	*Amylomyces rouxii*	Smith et al. (2021)
*Armillaria*		Smith et al. (2021)
	*Armillaria mellea*	Smith et al. (2021)
*Amylomyces*		Smith et al. (2021)
*Armillaria*		Smith et al. (2021)
	*Armillaria mellea*	Smith et al. (2021)
*Aspergillus*		Smith et al. (2021), Gandia et al. (2022)
	*Aspergillus nidulans*	Smith et al. (2021)
	*Aspergillus niger*	Smith et al. (2021)
	*Aspergillus oryzae*	Smith et al. (2021)
*Bjerkandera*		Gandia et al. (2022)
*Calocybe*		Stewart et al. (2021)
	*Calocybe gambosa*	Stewart et al. (2021)
*Calvatia*		Stewart et al. (2021)
	*Calvatia gigantea*	Stewart et al. (2021)
*Cerioporus*		Ross et al. (2020b), Smith et al. (2021)
	*Cerioporus squamosus*	Ross et al. (2020b)
*Ceriporia*		Smith et al. (2021)
	*Ceriporia lacerata*	Smith et al. (2021)
*Cerrena*		Gandia et al. (2022)
*Coprinus*		Smith et al. (2021)
	*Coprinus comatus*	Smith et al. (2021)
*Cordyceps*		Stewart et al. (2021)
	*Cordyceps militaris*	Stewart et al. (2021)
*Disciotis*		Stewart et al. (2021)
	*Disciotis venosa*	Stewart et al. (2021)
*Fibroporia*		Smith et al. (2021)
	*Fibroporia vaillantii*	Smith et al. (2021)
*Fistulina*		Smith et al. (2021)
	*Fistulina hepatica*	Smith et al. (2021)
*Flammulina*		Smith et al. (2021)
	*Flammulina velutipes*	Smith et al. (2021)
*Fomes*		Stewart et al. (2021), Szilvay et al. (2021), Gandia et al. (2022)
	*Fomes fomentarius*	Szilvay et al. (2021)
*Fomitopsis*		Smith et al. (2021), Gandia et al. (2022)
	*Fomitopsis officinalis*	Smith et al. (2021)
*Fusarium*		Smith et al. (2021), Stewart et al. (2021), Gandia et al. (2022)
	*Fusarium venenatum*	Stewart et al. (2021)
*Flypholoma*		Smith et al. (2021)
	*Flypholoma capnoides*	Smith et al. (2021)
	*Flypholoma sublateritium*	Smith et al. (2021)
*Ganoderma*		Ross et al. (2020b), Smith et al. (2021), Stewart et al. (2021), Szilvay et al. (2021), Gandia et al. (2022)
	*Ganoderma applanatum*	Ross et al. (2020b)
	*Ganoderma lucidum*	Ross et al. (2020b), Szilvay et al. (2021), Mueller et al. (2022)
	*Ganoderma oregonese*	Ross et al. (2020b)
	*Ganoderma resinaceum*	Ross et al. (2020b)
	*Ganoderma sessile*	Smith et al. (2021)
	*Ganoderma tsugae*	Ross et al. (2020b), Smith et al. (2021)
*Gibberella*		Smith et al. (2021)
*Grifola*		Stewart et al. (2021)
	*Grifola fondosa*	Stewart et al. (2021)
*Hericulum*		Stewart et al. (2021)
	*Hericulum erinaceus*	Smith et al. (2021), Stewart et al. (2021)
*Hymenoscyphus*		Raman et al. (2022)
	*Hymenoscyphus fraxineus*	Raman et al. (2022)
*Hypholoma*		Stewart et al. (2021)
	*Hypholoma lateritium*	Stewart et al. (2021)
*Hypsizygus*		Stewart et al. (2021)
	*Hypsizygus marmoreus*	Stewart et al. (2021)
	*Hypsizygus ulmarius*	Stewart et al. (2021)
*Inonotus*		Smith et al. (2021)
	*Inonotus obliguus*	Smith et al. (2021)
*Lactarius*		Smith et al. (2021)
	*Lactarius chrysorrheus*	Smith et al. (2021)
*Macrohyporia*		Gandia et al. (2022)
*Macrolepiota*		Smith et al. (2021)
	*Macrolepiota procera*	Smith et al. (2021)
*Morchella*		Smith et al. (2021), Stewart et al. (2021)
	*Morchella angusticeps*	Smith et al. (2021)
	*Morchella conica*	Stewart et al. (2021)
	*Morchella esculenta*	Stewart et al. (2021)
	*Morchella importuna*	Stewart et al. (2021)
	*Myceliophthora thermophila*	Smith et al. (2021)
*Myxotrichum*		Gandia et al. (2022)
*Neurospora*		Smith et al. (2021)
	*Neurospora crassa*	Smith et al. (2021)
*Penicillium*		Smith et al. (2021)
	*Penicillium camemberti*	Smith et al. (2021)
	*Penicillium chrysogenum*	Smith et al. (2021)
	*Penicillium rubens*	Smith et al. (2021)
*Perenniporia*		Gandia et al. (2022)
*Phellinus*		Gandia et al. (2022)
*Pholiota*		Stewart et al. (2021)
	*Pholiota nameko*	Stewart et al. (2021)
*Phycomyces*		Smith et al. (2021)
	*Phycomyces blakesleeanus*	Smith et al. (2021)
*Pleurotus*		Smith et al. (2021), Stewart et al. (2021), Szilvay et al. (2021)
	*Pleurotus djamor*	Smith et al. (2021)
	*Pleurotus eryngii*	Stewart et al. (2021)
	*Pleurotus ostreatus*	Smith et al. (2021), Stewart et al. (2021), Szilvay et al. (2021)
*Polyporous*		Stewart et al. (2021)
	*Polyporous squamosus*	Smith et al. (2021), Stewart et al. (2021)
*Psathyrella*		Smith et al. (2021)
	*Psathyrella aguatica*	Smith et al. (2021)
*Pycnoporus*		Gandia et al. (2022)
*Rhizopus*		Smith et al. (2021), Gandia et al. (2022)
	*Rhizopus microsporus*	Smith et al. (2021)
	*Rhizopus oryzae*	Smith et al. (2021)
*Schizophyllum*		Ross et al. (2020b), Gandia et al. (2022)
	*Schizophyllum commune*	Appels et al. (2020), Ross et al. (2020b), Smith et al. (2021)
*Sparassis*		Stewart et al. (2021)
	*Sparassis crispa*	Stewart et al. (2021)
*Streptomyces*		Smith et al. (2021)
	*Streptomyces venezuelae*	Smith et al. (2021)
*Stropharia*		Stewart et al. (2021)
	*Stropharia rugosoannulata*	Smith et al. (2021), Stewart et al. (2021)
*Thielavia*		Smith et al. (2021)
	*Thielavia terrestris*	Smith et al. (2021)
*Trametes*		Attias et al. (2020), Ross et al. (2020b), Gandia et al. (2022), Mueller et al. (2022)
	*Trametes versicolor*	Ross et al. (2020b)
	*Trametes pubescens*	Ross et al. (2020b)
	*Trametes ochracea*	Attias et al. (2020)
*Trichoderma*		Szilvay et al. (2021), Gandia et al. (2022)
	*Trichoderma reesei*	Szilvay et al. (2021)
*Tuber*		Stewart et al. (2021)
	*Tuber borchii*	Stewart et al. (2021)
*Tyromyces*		Gandia et al. (2022)
*Ustilago*		Smith et al. (2021), Stewart et al. (2021)
	*Ustilago esculenta*	Stewart et al. (2021)
	*Ustilago maydis*	Smith et al. (2021)

Upon critical evaluation of the patents, it must be recognized that the mere mention of certain species in the claims or description of the patents do not guarantee their effectiveness or compatibility with the production and application of mycelium materials. This information is only a starting point for a more in-depth analysis and comparison. Furthermore, given the large phylogenetic diversity of filamentous fungi (Hawksworth and Lücking, 2017), it could be envisaged that there is still an unexplored wealth of species with the potential to be used in the production of mycelium materials with different properties and unknown advantages. Besides investigating the diversity of natural strains, genetic engineering of already good performing strains can be another approach to improve material characteristics (Vandelook et al., 2021; Bayer et al., 2022).

#### 3.2.2 Fermentation techniques, apparatuses and systems

**FIGURE 2:**
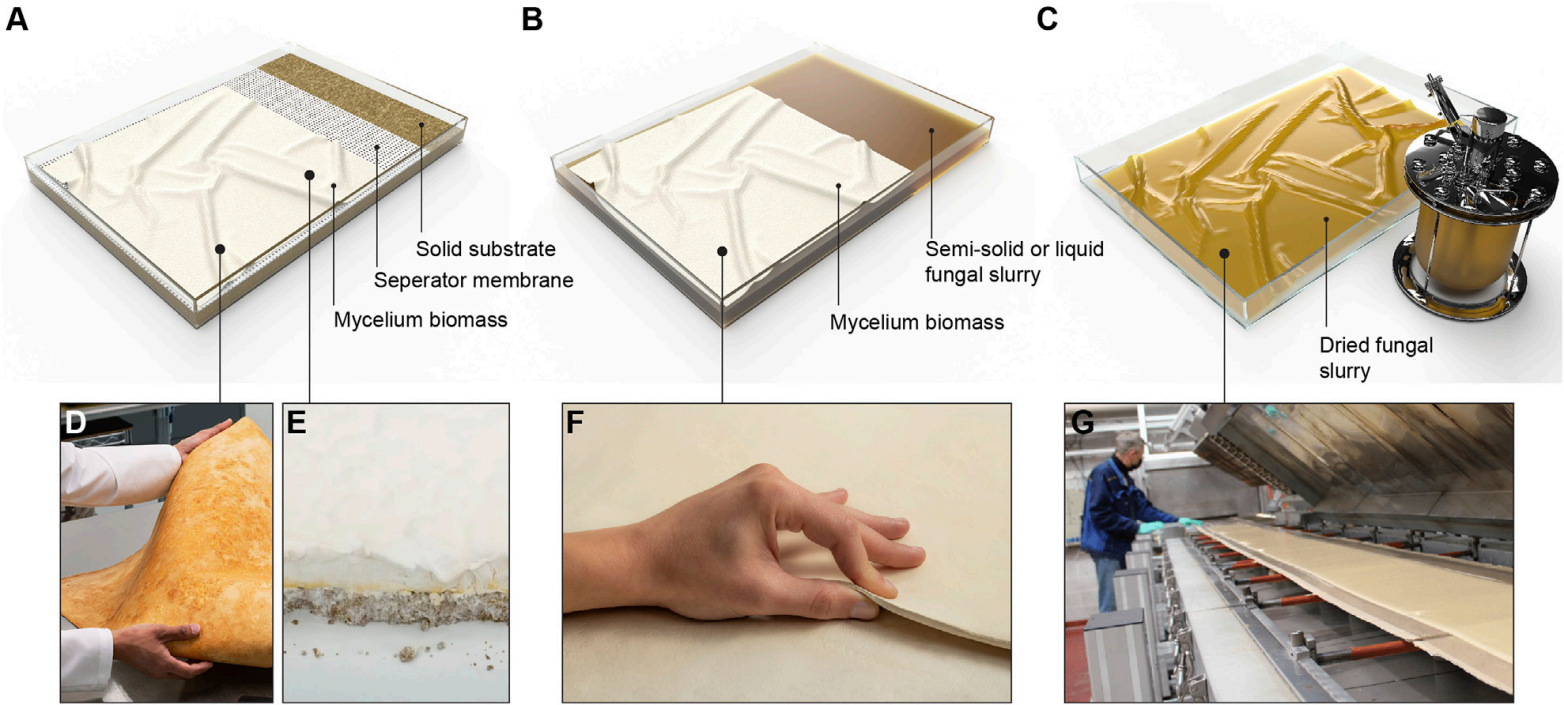
Three main fermentation techniques for fungal mats production. **(A)** Schematic visualization of solid-state surface fermentation. **(B)** Schematic visualization of liquid-state surface fermentation. **(C)** Schematic visualization of stirred submerged liquid fermentation. **(D)** Myco-leather called Reishi™ made by Mycoworks (United States). **(E)** Foam called Forager made by Ecovative (United States) **(F)** Raw 100% mycelium materials called ephea™, developed by SQIM/MOGU (Italy). **(G)** Continuous mycelium leather production at VTT Technical Research Centre of Finland.

There are three main strategies to generate fungal biomass intended for the application of leather-like mycelium materials: solid-state surface fermentation (SSSF), liquid-state surface fermentation (LSSF), and stirred submerged liquid fermentation (SSLF). While SSSF and LSSF fermentation techniques allow the mycelium to be grown as one whole sheet, SSLF results in a lower-cohesion broth with slurry or pellets that requires further processing to be formed as a coherent piece of material. SSSF involves introducing a fungal organism to a solid growth substrate, allowing for the development of mycelial tissue at the surface of the substrate (Figure 2A). This method often utilizes lignocellulosic substrates, similar to those utilized in the cultivation of edible mushrooms, due to their affordability and availability. LSSF can either use lignocellulosic fibers mixed into a liquid broth or uses a completely dissolved nutrient solution, resulting in the growth of a fungal tissue at the liquid-air interface in a static setup (Figure 2B). SSLF entails the cultivation of submerged fungal biomass in a liquid medium using a bioreactor, bubble column reactor, or shake flask setup (Figure 2C).

##### 3.2.2.1 Solid-state surface fermentation

Solid-state surface fermentation (SSSF) is widely used for cultivation mycelium leather-like materials. Companies such as Ecovative, Mycoworks, and Mycotech have patented various variations of this method (Bentangan et al., 2020; Ross et al., 2020b; Greetham et al., 2022; Kaplan-Bie et al., 2022). In SSSF, a flat or foam-like mycelium layer grows on top of a solid lignocellulosic substrate (Figure 2A). The pure fungal biomass is easily separated from the substrate by cutting off the top mycelium layer. The remaining substrate can be used to produce lignocellulosic mycelium composites for different applications. The substrate is either pre-grown with a fungal inoculum in bags or introduced directly into an enclosed mold for incubation to produce a pure mycelium layer.

**FIGURE 3:**
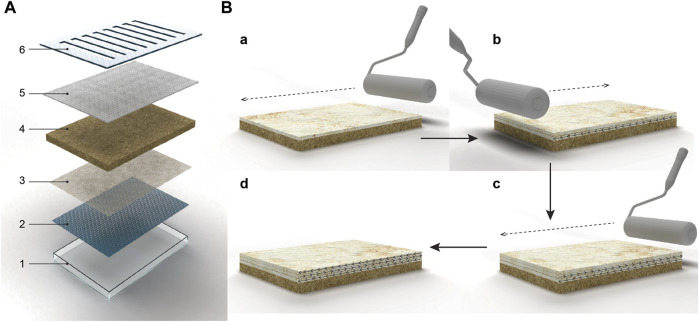
Solid-state fermentation strategy that involves embedding a porous intermediate membrane, made of a fungus-resistant polymer, on top of the solid substrate. **(A)** Illustration of a mycelium growth bed comprising a tray (1), a conveying platform (2) that is configured to fit within the tray, a perforation layer with many pores (3), a mass of mycelium substrate inoculated and colonized (4), a porous material (5) placed on top of the mycelium substrate. The tray can be closed with a lid (6) containing openings for a porous material. Reworked from Figure 1 in patent of (Ross et al., 2020a) **(B)** Illustration of a sequence of manipulations of mycelium materials by flattening the grown hyphal network **(a)** Dense network of hyphae grown on a nutritional substrate being rolled flat in one direction. **(b)** The second layer of regrown hyphae being flattened in the opposite direction of the first layer. **(c)** Third layer of regrown hyphae being flattened on top of the first and second flattened layer. **(d)** Hyphae being flattened in multiple directions. Reworked from Figure 3 in patent of (Ross et al., 2020b).

The patent of Ross *et al.* (Mycoworks) describes a strategy that involves embedding a porous intermediate membrane, made of a fungus-resistant polymer, on top of the solid substrate (Ross et al., 2020b) (Figure 2D). The hyphae grow through this intermediate layer, allowing easy separation of the fungal material from the nutritive substrate. The growth direction of the organism can potentially be guided by electrical actuation (Ross et al., 2020b). In the SSSF process developed by Mycoworks (Figure 3A), an intermediate layer is placed at the bottom of the mold. The inoculated substrate is then packed on top, and mechanical pressure is applied to flatten the layer. The mold is covered and incubated for 2–4 days to facilitate the growth of the fungus inside the substrate and through the intermediate layer. The solid substrate is then removed from the mold and the mold is then flipped, placing the intermediate layer on top. The substrate block is returned to the mold and incubated, stimulating hyphal growth away from the substrate and into the air. Upon the visible growth of the mycelium through the intermediate layer, a contaminant-free cellulose-based textile (e.g., cotton) is placed on top of the hyphae to form a composite material with improved material properties. Optimal growth conditions include a humid environment (humidity range of 20%–100%), high oxygen content, a temperature of 22°C–25°C, and total darkness. The fungus continues to grow over the next 2 weeks, during which daily manipulations, such as flatting the mycelium sheet with a rolling pin in different directions, are performed (Figure 3B). This process can be repeated with multiple layers to enhance bonding. Once the desired thickness is reached, the intermediate layer is delaminated from the solid substrate, followed by several post-growth processing steps.

The method developed by Bentangan *et al.* (Mycotech) involves growing a mycelium layer on a solid substrate, but without utilizing an intermediate layer to separate the mycelium from the substrate or rolling the hyphae in different directions (Bentangan et al., 2020). As a result, residual substrate particles can remain attached at the bottom of the mycelium sheet, which must be cleaned with a brush. To preserve the mycelium tissue and prevent rot, it is treated with salt and boiled in water (Bentangan et al., 2020).

**FIGURE 4:**
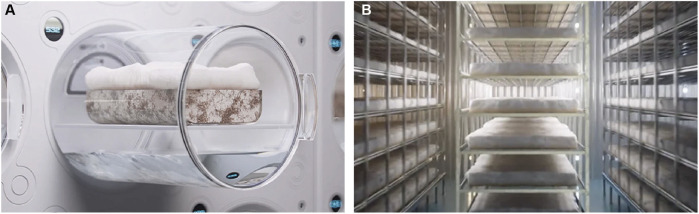
**(A)** Illustration of abundant aerial mycelium growth on top of a solid substrate, under high CO_2_ concentration, directed airflow and micro-droplet deposition **(B)** Illustration of a shelf system for scaling aerial mycelium foam called AirMycelium™, developed by Ecovative Design.

The fermentation strategy developed by Kaplan-Bie *et al.* and Greetham *et al.* (Ecovative, 2023) involves stimulating abundant aerial mycelium growth on top of a solid substrate, under high CO_2_ concentration, directed airflow and micro-droplet deposition (Figure 4A) (Greetham et al., 2022; Kaplan-Bie et al., 2022). Typically, the CO_2_ concentration is kept at 5%, the temperature can fluctuate between 30°C and 32°C, the airflow rates vary between 2.8–10 m^3^/min, and the mean mist deposition rate is less than or equal to about 5 microliter/cm^2^/hour (Winiski et al., 2022). Under these conditions, the undifferentiated mycelium grows into the void space, which is then separated from the substrate and dried. This results in a thick foam-like mycological biopolymer composed entirely of fungal mycelium (Figure 2E). Open trays containing the nutritive substrate and organism are placed in an incubation chamber (Figure 4B) where lateral or perpendicular airflow with high carbon dioxide content is directed above the trays. Specific airflow velocities can produce different aerial mycelium densities (Kaplan-Bie et al., 2022). A mist containing solutes (e.g., minerals, proteins or carbohydrates) is circulated through the incubation chamber and deposited onto the growing tissue. The controlled environment established in the process allows the growth and development of the mycelium to be influenced by applying various morphological modifiers. Small variations in parameters such as relative humidity and airflow speed can noticably affect the properties of the resulting fungal biopolymer. For example, adjusting the relative humidity from 99% to less than 98% for 4–72 h induces densification of the fungal tissue, which can then be grown in a less dense manner by raising the humidity back to 99%, resulting in a multi-layered density biopolymeric foam. The patent also states that the tensile strength of the mycelium material increases with an increased airflow speed (Kaplan-Bie et al., 2022). Similar to Ross *et al.*’s invention, a non-substrate porous layer can also be placed on top of the substrate to reinforce the mycelium material or to facilitate the removal of the mycelium tissue. After being removed from the substrate, the material is further processed to improve density and/or mechanical strength.

##### 3.2.2.2 Liquid-state surface fermentation

LSSF, as described by Gandia et al. (2022) (Mogu), begins by inoculating and growing a filamentous fungus on a sterilized or pasteurized solid lignocellulosic substrate, which may be supplemented with seeds, seed flour, starch powder, and/or minerals (Gandia et al., 2022). A liquid medium, such as Malt Extract Broth (MEB), Malt Yeast Extract Broth (MYEB), or Potato Dextrose Broth (PDB), is added to the solid substrate at a ratio of 2%–5% medium per total weight of solid substrate. The incubation is performed in static and aerobic conditions, in the dark and at temperatures between 20°C and 30°C. This growth phase continues until the fungus fully colonizes the substrate, typically taking 5–15 days.

**FIGURE 5:**
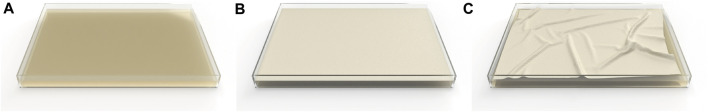
Illustration of the different steps in the liquid state fermentation production process. **(A)** Living fungal slurry is poured into a flat container. **(B)** Fungal mat grows on top of the slurry during the incubation. **(C)** The fungal mat is harvested by peeling it off the digested slurry underneath. Reworked from Figure 3 in patent of (Gandia et al., 2022).

In a second phase, a homogeneous, viscous fungal slurry is prepared by blending the colonized lignocellulosic substrate with sterile water (Gandia et al., 2022). The colonized medium is mixed with water at a ratio of 2 g of substrate per 10 mL of water. The resulting semi-solid substrate is then placed in a flat container (Figure 5A) and incubated until a fungal tissue forms on the top surface of the slurry (Figure 2C; Figure 5B). Incubation is carried out in static and aerobic conditions at a constant temperature. Depending on the fungal species, a constant CO_2_ concentration of 2000–2,500 ppm maintained. After the mycelium reaches the desired thickness and density, typically after 10–18 days, it can be harvested by peeling it off the digested slurry underneath and rinsing its surface with water (Figure 2F; Figure 5C).

Fungal sheets can also be combined and re-incubated for at least 2 days to form a multilayer fungal material. During this process, new hyphae will grow and form a natural bond between the different layers. The mechanical properties of the mycelium material can be enhanced by adding a porous material, such as a layer of fibrous textile (e.g., hemp, linen, or cotton) or a polymer, on top of the surface. This allows the fungi to grow into the layer without digesting it. Additionally, including foaming agents like carrageenan (0.1%–1%) or albumin (0.1%–1%) in the creates a foamy substance with many air bubbles, which benefits the growth of the mycelium. At this stage, other additives such as cellulose acetate, chitin or chitosan, corn zein and starch, sucrose, dextrose, malt extract, or molasses may also be added. Finally, the fungal material undergoes further post-growth processing.

##### 3.2.2.3 Stirred submerged liquid fermentation

The SSLF strategy, described in a patent by Szilvay et al. (2021) (VTT), involves cultivating a fungal species in a stirred submerged liquid suspension (Figures 2C, G). By allowing the organism grow inside a bioreactor, bubble column reactor, or shaking flask setup (at 200 rpm), a large amount of fungal biomass can be produced (Szilvay et al., 2021). An important difference between SSLF and the other fermentation techniques is the requirement of active stirring to submerge the organism throughout the cultivation process, in contrast to the more passive cultivation strategy used in static surface fermentation that requires minimal energy input.

It is preferable to use water-soluble nutritional sources to create a homogenous liquid medium for an efficient stirring, although small particles could also be used to offer an anchoring point for the fungal organism. One example of added small particles to the stirred solution are nanocellulose fibrils (Szilvay et al., 2021). This method, previously reported in scientific literature in the context of aerogel production by incubation of a fungal species with nanocellulose (Attias et al., 2020) can be combined with polymers, fibers, and coloring agents during or at the end of the fermentation process (Szilvay et al., 2021).

After the SSLF process is completed, the cultured mycelium is filtered out of the liquid growth medium, homogenized and washed with water. Crosslinking agents, such as citric acid, can be added, as well as plasticizers, either during stirring or after the washing step. The processed mycelium is then dried, optionally through a heat treatment. Mycelium sheets can be produced using lyophilization and/or vacuum filtration through membranes (Appels et al., 2020; Attias et al., 2020). Semidried films are carefully transferred to a frame for constrained drying to prevent shrinkage and wrinkling (Attias et al., 2020) or covered with cellophane on a flat surface (Appels et al., 2020).

#### 3.2.3 Post-growth plasticizing and crosslinking strategies

If left untreated, mycelium materials will become stiff and brittle when fully dried (Appels et al., 2020). Therefore, a plasticizing agent is applied to keep the sheets flexible. Typically, any kind of polyols can be used to plasticize mycelium-based materials (composed of glucan, chitin and/or chitosan biopolymers), such as propylene glycol (Ross et al., 2020b). Alternatively, the plasticizer can also be selected from sugar alcohols, epoxy esters, ester plasticizers, glycerol esters, phosphate esters, terephthalates, leather conditioners, acetylated monoglycerides, alkyl citrates, epoxidized vegetable oils, methyl ricinolate, or other common polymers plasticizers (Szilvay et al., 2021).

Hyphal cell walls can be chemically crosslinked, which increases the strength and stiffness of the material while preserving its extendibility. For example, citric acid was found to react with the glucan hydroxyl groups present in the cell wall, causing crosslinking between the cell walls of the neighboring hyphae crosslink together (Szilvay et al., 2021). The crosslinking agents can be polycarboxylic acid, tricarboxylic acid, dicarboxylic acid, glutaraldehyde, and tannin (such as pyrogallols and glutaraldehyde). In another embodiment, the crosslinking target is an amine group present on the deacetylated chitin polymer (chitosan) (Ross et al., 2020b). Enzymes potentially expressed and secreted by the fungi during the cultivation process, such as oxidase and oxidoreductase, or laccase and tyrosinase, can also be used to crosslink tannins, lignin or vanillin into the mycelium cell wall (Szilvay et al., 2021). Additionally, fungal strains producing crosslinking agents such as dicarboxylic and tricarboxylic acid can be used. Crosslinking can also be achieved through heat treatment ranging from 90°C to 150°C (Szilvay et al., 2021), for example, by pressing the mycelium sheets between hot plates.

An effective crosslinking strategy for mycelium materials, developed by Ecovative (2023), Kaplan-Bie (2018), involves the combination of an organic solvent solution (e.g., alcohol), a calcium chloride solution, and a phenol/polyphenol solution. The organic solvent enables penetration of the material, rinses away extracellular materials, denatures proteins and partially deacetylates chitin. The addition of phenols to the treatment solution acts as crosslinking agents, creating covalent bonds between the primary amine of deacetylated chitin (chitosan) and amines- and hydroxyl-groups of amino acid residues, improving the mechanical properties of the final material. The use of salt as a humectant and antimicrobial agent ensures functional preservation of the material and provides added protection against microbial growth. The addition of methanol and calcium chloride further deacetylates chitin and mediates bond formation (Kaplan-Bie, 2018). In water, the salt can form ionic bonds with the same functional groups, further reinforcing the structure of the mycelium material. The steps in this technique are as follows (Kaplan-Bei, 2018). First, a solution of 10 g/L tannic acid powder and water is prepared, into which the mycelium material is immersed for 7 days. The mycelium material is then placed in a bath of 150 g/L salt and 100% alcohol (e.g., isopropyl, ethanol, methanol) for up to 7 days before being repeated. The material is then taken from the water and pressed between rollers. It is immersed in 100% alcohol for 1 day before being pressed again. After that, the tissue is allowed to air dry before being treated with a plasticizer, such as a 20 g/L glycerin or sorbitol solution in water, to achieve the desired softness and flexibility (Kaplan-Bie, 2018).

Mycoworks’ crosslinking strategy primarily acts on chitosan, which has easily reactive primary amine groups that form amide bonds during crosslinking (Deeg et al., 2017; Chase et al., 2019). To partially deacetylate the chitin within the fungal material and chitin nanowhiskers, they are submerged in an aqueous solution of 40% by weight of sodium hydroxide at 80 °C for a time period ranging from one minute to ten hours (Chase et al., 2019). This process can achieve a desired degree of acetylation from 1% to 50%. After this step, the fungal material is impregnated with chitin nanowhiskers by soaking and agitation. The addition of nanoparticles greatly enhances the performance of chitinous structures. Chitin nanowhiskers can be used to crosslink the primary amine groups in chitosan and the blocked isocyanate crosslinker hexamethylene-1,6-di-(aminocarboxysulfonate) (Araki et al., 2012). The nanowhiskers fill the gaps between the cell wall chitosan chains, forming a nanocomposite which is further strengthened through crosslinking (Deeg et al., 2017). The resulting chitosan-nanowhisker material has improved elastic modulus and tensile strength (Araki et al., 2012). Then, to crosslink the fungal material with a strength bearing element/backing (such as cellulosic textiles), commercially available genipin powder is dissolved in acetic acid (Chase et al., 2019). The naturally-derived cross-linking agents genipin is particularly promising as it is relatively efficient and has been extensively studied in relation to its cross-linking properties with chitosan (Mi et al., 2000). However, a side effect of using genipin is that it can cause a blue discoloration of materials (Deeg et al., 2017). The functional groups of genipin responsible for its cross-linking capabilities are the ester and the third carbon in the six-membered dihydropyran ring (Mi et al., 2000; Butler et al., 2003). Both of these functional groups react with the primary amine group in chitosan, forming connections between two chitosan chains (Mi et al., 2000; Butler et al., 2003). The resulting genipin mixture is then mixed with an undisclosed solution that has a pH between 2 and 3 (Chase et al., 2019). This second mixture is applied to the fungal material at a specific rate (ranging from 0.05% to 4% of the weight of the genipin polymer) to create a genipin-fungal mixture. This mixture is then incubated at 25°C for 40 min to several hours with agitation. Finally, the fungal material is rinsed with water.

#### 3.2.4 Coating processing techniques

The physical properties of pure flexible mycelium material are sometimes insufficient and must be improved for diverse types of applications. Various coating techniques borrowed from the traditional leather and textile manufacturing industry can enhance the functional properties of mycelium materials. Coating agents such as dyes, resins, oils, paraffins, and polymers of natural or synthetic origins can be applied to the mycelium material using methods like air spray, curtain coating, or dip coating.

One technique that creates a protective barrier and simultaneously improves the mechanical properties of mycelium-based materials is the lamination process, which involves applying a thin polymeric film. Poly(L-lactic) acid (PLA), a sustainable polyester produced through microbial fermentation, is one example of a polymer used in this process. Mycoworks (Scullin et al., 2020) has developed a lamination method that applies heat and pressure to bind a PLA film onto the surface of the mycelium-based material. This increases the strength and durability while preserving the flexibility and (bio-)degradability (Scullin et al., 2020).

In addition to these traditional coating techniques, Mycoworks and Mogu utilize a biodegradable polymer eluted in a mixture of water and an organic solvent (Scullin et al., 2020; Gandia et al., 2021a). The organic solvents utilized in these bio-based polymers can include alcohol, ketones, ethers, alkanes, cyclic ethers, glycol ethers, and phenylated solvents. The polymer is dispersed in water at a concentration ranging from 0.1% to 50% (Scullin et al., 2020). Water carries the polymer into the fungal matrix to fortify the hyphae, and improve abrasion resistance, colorfastness to crocking, dye transfer and water resistance (Scullin et al., 2020). Vegetable oils (soybean, sunflower, corn) can also be used as natural bioresources for the production of bio-based polyurethanes (Gandia et al., 2021a). Alternative biomass resources, such as fungal polyols, chitin, and glucans can be employed as well. Coatings typically include crosslinking agents and various additives such as surfactants, antifoaming agents, anti-gelling agents, anti-oxidation agents, thickening agents, plasticizers, flame retardants, pigments, and fillers (Gandia et al., 2021a).

The coating application process can be performed in multiple steps using spray or roll-transfer methods (Scullin et al., 2020). An initial layer of a polyurethane or acrylic containing medium is applied to promote adhesion. Subsequently, additional layers containing color pigments, acrylics, silicones, resins, or polyurethanes are applied and dried. This process is followed by a heating and pressing, which can be achieved using a heated roller. The final step is drying the material between 50°C and 150°C to remove moisture.

### 3.3 Material properties

Currently, there is a need for a standardized characterization study gathering all available mycelium-based leather-like materials from commercial players in the field. While a few independent third-party characterization results have been made publicly available, testing standards can still differ between different countries (ASTM *versus* ISO) and among different compositions and finishes of leather-like product. Relevant material properties of leather, beside the thickness (ASTM D1813, ISO 2589) and apparent density (ASTM D2346, ISO 2420) are tensile strength and percentage elongation (ASTM D2209 and D2211, ISO 3376), tear strength (tongue tear) (ASTM D4704, ISO 3377), abrasion resistance (ASTM D7255, ISO 17076) and colorfastness when exposed to diverse conditions (wash, seawater, alkali, acid, wet-/dry crocking, perspiration, etc.). The testing and standardization process for these new materials is further complicated by the various types of ingredients and additional materials used in combination with mycelium biomass to achieve a finished leather-like product. Consequently, different standard tests are needed to accurately characterize the materials, considering that mycelium is almost always combined with different elements to fulfil its functional role as leather-like material.

The complexity of characterization, arising from the diverse array of material compositions, offers the advantage of tailoring the material properties specific to different applications. Mycelium-based materials serve as a basic platform that allows for tuning and engineering a multitude of characteristics and properties depending on the users’ requirements. Customizable tensile strength, density, and fiber orientation are considered key selling points of these materials. Another superior advantage over traditional leather is the scrap-less confection process enabled by the material’s homogeneity and ability to customize order size. Currently singular pieces of up to 60 m × 4 m are achievable (Ecovative, 2023).

For publicly reported values of different properties of commercial leathery mycelium materials, we refer to the work of (Jones et al., 2020a; Vandelook et al., 2021).

## 4 Perspectives and conclusion

This patent overview summarizes the most recent leather-like mycelium material patent publications and indicates that this field is currently experiencing a rapid expansion. There has been a significant rise in patents published since the end of 2019. Almost 80% of the granted patents originated from US applications. The examination of patents provides a frame of the processing methods and fermentation techniques that are being focused on for valorization purposes. Three main methods for growing fungal biomass were derived from the patents: solid-state surface fermentation, liquid-state surface fermentation, and stirred submerged liquid fermentation. In short, filamentous fungi can be grown on the surface of a solid growth medium or on the surface of a liquid medium. They can also be grown fully submerged in a liquid medium using a bioreactor or shaking flask setup. Additionally, different patents describe how hyphal cell walls can be chemically crosslinked, which often increases the material’s strength and stiffness while preserving its extendibility. Finally, a variety of coating products can be used to cover the mycelium material, including spraying or laminating thin (water-based, bio-based) polymeric films.

In order to maintain a competitive edge in the early stages of commercialization, it became apparent throughout the patent search that applicants frequently employ vague and generic descriptions of their innovations. One notable limitation of the patents reviewed in this study is the absence of quantitative data and references to internationally recognized standards such as ISO (International Organization for Standardization). So far, the field of mycelium materials is still young, and it is clear from reviewing patent literature that private companies have been investing more time and effort into advancing research and stimulating innovation. Unfortunately, this means that much of the produced data is restricted or of limited access, which can have an inefficient effect on ongoing research. The lack of specific numerical values and standardized testing procedures makes it challenging to directly compare and evaluate the results reported in different patents. The absence of quantitative data hinders a comprehensive understanding of the performance and characteristics of the described mycelium materials and their applications. There’s a need for more publicly funded research at the different levels, encompassing the production of mycelium materials to the consumer experience, and identify any negative or harmful elements or processes that could impact the environment or human health.

For instance, according to a recent study funded by MycoWorks, the growth process of Ecovative, which involves pumping large amounts of CO_2_ into the aerial mycelium growth chamber, is likely to have a very high carbon footprint because it burns fuels at the source of CO_2_ production and then releases that CO_2_ into the atmosphere as the mycelium grows (Williams et al., 2022). Further research is required on the effects of mycelium material production on climate change and carbon footprint to enhance production methods and promote technical choices that will benefit large-scale facilities. Furthermore, most mycelium products now available on the market feature some sort of PU coating that ranges in thickness from 10 to 500 μm. These synthetic polymers are added to protect and or reinforce the mycelium material, but hinders the biodegradability in a natural environment. It will be necessary to continue advancements in sustainable coatings to get beyond the drawbacks of employing non-sustainable synthetic coatings.

In conclusion, based on the recent increase in patent applications, it is reasonable to expect substantial breakthroughs and a further increase in the number of patents on the topic of leather-like mycelium materials in the following decade.

## Data Availability

The original contributions presented in the study are included in the article/Supplementary Material, further inquiries can be directed to the corresponding authors.
